# Health promotion targeting older people

**DOI:** 10.1186/s12913-016-1514-3

**Published:** 2016-09-05

**Authors:** Stanisława Golinowska, Wim Groot, Petra Baji, Milena Pavlova

**Affiliations:** 1 Faculty of Health Sciences, Department of Health Economics and Social Security, Institute of Public Health, Jagiellonian University Collegium Medicum, ul. Grzegórzecka 20, 31-531 Krakow, Poland; 2 Department of Health Services Research, CAPHRI, Maastricht University Medical Center, Faculty of Health, Medicine and Life Sciences, Top Institute Evidence-Based Education Research (TIER), Maastricht University, PO Box 616, 6200 MD Maastricht, The Netherlands; 3 Department of Health Economics, Corvinus University of Budapest, Fővám tér 8, 1093 Budapest, Hungary; 4 Department of Health Services Research, CAPHRI, Maastricht University Medical Center, Faculty of Health, Medicine and Life Sciences, Maastricht University, PO Box 616, 6200 MD Maastricht, The Netherlands

Health is determined by behavior in many ways. The most well-known types of health-related behavior – smoking, alcohol use and exercise/eating habits – are only a selection of the behavioral aspects of health. A healthy lifestyle can be promoted by various means, ranging from educational and counseling programs to financial incentives for a healthy lifestyle. These interventions are further offered in various ways, ranging from general legislative measures to programmatic interventions. Health promotion interventions can take different forms, from small projects to large national programs. They can be funded and organized by donations from individuals or NGOs, or through taxation by national governments. In short, health promotion is typified by heterogeneity in every conceivable aspect.

Health promotion is meant for the entire population. If a specific group within a population is singled out as the recipient of health promotion interventions, it is because of a valid reason, such as epidemiological concerns or preferences in social policy (e.g. measures targeting vulnerable or disadvantaged groups). This explains the focus of many health promotion activities on youth, citizens of big cities, workers in certain industries or occupations.

The elderly have long been neglected as the addressee of health promotion activities. The need to promote health among older people was first highlighted in the 1990s [1]. Before that, it was commonly assumed that the older generations were not a good target for health promotion as it was thought it was too late to change their lifestyle. Requiring the elderly to radically change their diet and start exercising was perceived as disturbing to their peace and wellness. Therefore, it was only after 2001, when WHO experts unanimously stated the importance of a healthy lifestyle at every stage of life, health promotion measures targeted to the elderly started to grow in numbers. Evidence has shown that exercising, quitting smoking and limiting alcohol consumption, participating in learning activities and integrating in the community can help to inhibit the development of many diseases and prevent the loss of functional capacity, thus improving quality of life and lengthening life expectancy. Most of these health promotion activities among the elderly focus on the relatively younger seniors. Within the group of those aged 85+, the emphasis is more on appropriate medical attention from physicians and care givers rather than on their health behavior.

Health promotion targeted to older people differs significantly from that addressing younger generations. This partly stems from the fact that the health of older people is generally less than perfect [2]. Seniors are more likely to be suffering from chronic conditions and multi-morbidities, and their functional capacity is frequently limited [3]. This implies that the health promotion programs for the elderly have to account for these limitations in health and daily activities, and require more involvement of professional health promoters and more individualized approaches. The prevalence of certain lifestyle issues is also higher among the elderly. Elderly people are – for example – more likely to suffer from loneliness and social isolation. Also, because of their relatively shorter remaining life expectancy, the focus is more on health promotion activities that yield immediate effects. That is why health promotion programs for older people in European countries are mainly implemented at the local level by primary health care providers and nurses, and by NGOs, self-governing public authorities and voluntary organizations. These programs sometimes lack sustainable sources of funding.

Health promotion strategies for the elderly generally have three basic aims: maintaining and increasing functional capacity, maintaining or improving self-care [4], and stimulating one’s social network [5]. The idea behind these strategies is to contribute to a longer, independent and self-sufficient quality of life [6]. It should be noticed that there is an additional objective to be considered: the significance of social participation and integration of the elderly to maintain quality of life at old age [7]. There is ample evidence to support the claim that social bonds and social activities, e. g. continuing professional work, learning activities later on, participation in cultural events and social work and maintaining a social network are essential for healthy aging [8, 9]. For this, health promotion focusses on the inclusion of seniors into the social activities in the community. These measures are not always formal and do not always need direct external financing but they frequently require an appropriate social and transport infrastructure.

**Fig. 1:**
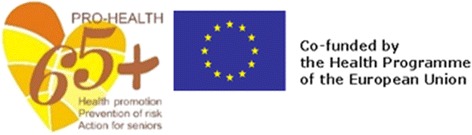
Project ProHealth 65+

This special issue of BMC Health Services Research contains a collection of research articles that focus on the funding, organization, coordination and cost-effectiveness of health promotion activities for older persons. The articles arise from the project ProHealth 65+ (http://pro-health65plus.eu/), which has received funding from the European Union, in the framework of the Health Programme (2008–2013), see Fig. 1. ProHealth 65+ is focused on health promotion and prevention of health risks among seniors. The project seeks to develop new knowledge on proven and cost-effective methods of health promotion targeting elderly population. The analysis of funding, organization and coordination of health promotion activities for older persons is also a key project objective. The project objectives underline the articles included in this special issue.

The articles present review-based analyses of public institutions, their legal basis, sources and methods of financing, and ways of conducting cost-effective interventions in the area, from a cross-country perspective with an emphasis on European countries. The studies are aimed to enrich our knowledge on the possibilities and barriers related to promoting health among older persons, and to provide insights on policies for healthy aging.
